# Nanorefractive index transducer using a ring cavity with an internal h-shaped cavity grounded on Fano resonance

**DOI:** 10.1371/journal.pone.0301007

**Published:** 2024-05-17

**Authors:** Shuwen Chang, Shubin Yan, Feng Liu, Jin Wang, Yuhao Cao, Biyi Huang, Chuanhui Zhu, Taiquan Wu, Yifeng Ren

**Affiliations:** 1 School of Electrical and Control Engineering, North University of China, Taiyuan, China; 2 School of Electrical Engineering, Zhejiang University of Water Resources and Electric Power, Hangzhou, China; 3 Joint Laboratory of Intelligent Equipment and System for Water Conservancy and Hydropower Safety Monitoring of Zhejiang Province and Belarus, Hangzhou, China; Universiti Brunei Darussalam, BRUNEI DARUSSALAM

## Abstract

Building on the Fano resonance observation, a new refractive index transducer structure at the nanoscale is proposed in this article, which is a refractive index transducer consisting of a metal-insulator-metal (MIM) waveguide structure coupled with a ring cavity internally connected to an h-shaped structure (RCIhS). Using an analytical method based on COMSOL software and finite element method (FEM), the effect of different geometric parameters of the structure on the trans-mission characteristics of the system is simulated and analyzed, which in turn illustrates the effect of the structural parameters on the output Fano curves. As simulation results show, the internally connected h-shaped structure is an influential component in the Fano resonance. By optimizing the geometrical parameters of the structure, the system finally accomplishes a sensitivity (S) of 2400 nm/RIU and a figure of merit (FOM) of 68.57. The sensor has also been demonstrated in the realm of temperature detection, having tremendous potential for utilization in future nano-sensing and optically integrated systems.

## Introduction

Surface plasmon polaritons (SPPs) [1, 2] are electromagnetic waves characterized by propagation along the surface of a metal-dielectric medium and exponential attenuation in the azimuth perpendicular to the surface direction. SPPs are formed due to the mutual intervention of random electrons together with incoming photons, providing a powerful breakthrough from the conventional diffraction limit of optics and enabling manipulation and transmission of photons in the sub-wavelength scale [3, 4]. For this property of SPPs, in which many novel optical devices can be integrated at the nanoscale, more and more waveguide structures have been proposed by re-searchers, e.g., metal nanoparticle arrays, hybrid Bragg waveguides, metal nanowires, V-groove waveguides, and metal-intermedium-metal (MIM). One of these is that electromagnetic waves propagate in MIM waveguide structures with lower losses, longer propagation distances and higher integration suitability. Hence, the MIM waveguide structure can efficaciously exclude the appearance of leakage and radiation modes and has superior local amplification capabilities [5, 6].

MIM optical waveguide consists of a three-layer structure fabricated from a metal-dielectric material composite, which functions as an important carrier for optical wave transmission, generating surface equipartition excitations and imposing certain constraints on them [7]. Typically, gold and silver are considered to be the metallic materials for MIM waveguides, and insulators or semiconductors are used as dielectric materials. MIM waveguides are capable of spawning SPPs on metal-dielectric surfaces, and when the thickness of the dielectric layer is small, the two SPPs generated couple to each other, and a large amount of energy can be generated in a small area by the ability of the MIM waveguide to confine the SPPs. Most of the optical energy is captured in the intermediate dielectric layer, which is well suited for photonic devices thanks to the long transmission distance of the MIM waveguide. The advantages of MIM waveguides can be summarized as simple structure, simple excitation, and propagation characteristics with sub-wavelengths, etc., compared with other surface-isolated excitations waveguide structures such as dielectric-metal-dielectric waveguides, V-slot waveguides, and hybrid Bragg waveguides. Furthermore, the MIM waveguide structure has better inclusiveness for the wavelength of SPPs, which makes further miniaturization of photonic devices possible.

Fano Resonance is a special optical phenomenon whose spectrum exhibits a sharp unsymmetrical linear shape. Fano resonance has considerable opportunities for application, and has been demonstrated in polymers, photonic crystals, and novel plasmonic structures, and has been widely used in slow light devices, filters, and sensors. Fano resonance is commonly induced by the interference between continuous broad-band modes and discrete narrowband modes, where the incident light directly energizes the continuous broadband modes, and the dipole portion of the metal waveguide structure resonates with the incident light field coupling to produce a large linewidth. Discrete narrow-band modes are individually stimulated by broad-band modes, which have comparatively narrow linewidths because they do not radiate energy outward and can store the energy of SPPs for long periods of time. The Fano resonance curve is extremely sharp and unsymmetrical, and it is suitable for distinguishing subtle translations of the curve. When the architectural parameters of the MIM wire guide and resonant chamber are continuously varied, the Fano resonance curve can be optimized to obtain transducers with better performance [8]. Generally speaking, the Fano resonance profile differs greatly when the architectural parameters of the system or the refractive index of the environment are modified. Consequently, the Fano resonance profile properties and MIM waveguide structural properties can be efficiently integrated and manipulated to achieve better performance of photonic devices in the sub-wavelength structural range.

The fact that various coupled resonant cavity structures based on MIM wave-guides have been suggested in recent years reflects the increasing recognition of this direction by researchers. MIM cavity waveguide configurations based plasma sensors are commonly used for refractive index sensing as their feedback characteristics are small changes in the surrounding material [9]. Resonator-built cavities and different geometries play a crucial role in designing better light-matter interactions in plasma sensing systems. In recent years, several MIM waveguides with different resonator modes have been investigated, such as circular/rectangular rings, bow-tie shaped cavities, toothed cavities, X-shaped, U-shaped, B-shaped, T-shaped, M-shaped, and bonded resonant cavities, elliptical trapezoidal cavities, full grating runway cavities, short truncated lines coupled to square cavities, metal nanorods in hexagonal configurations, and so on [10]. Currently, numerous devices based on Fano resonance for MIM waveguide-coupled resonant cavities have been proposed, comprising optical beam splitters [11], filters [12–14], directional couplers [15], optical switches [16, 17], Bragg reflectors [18, 19], and transducers [20–25], among others. Using surface equipartition excitations as the basic research theory and Fano resonance as the prominent research phenomenon, a nanoscale refractive index sensor structured as an annular cavity internally connected with an h-shape is proposed in this article. The proposed system is investigated using COMSOL software, and the transmission spectra and the electric and magnetic fields are analyzed theoretically, and the causes of the Fano resonance and its effects towards the transmission features of our system are discussed theoretically as well. According to the results, the geometrical parameters of the structure have a great impact upon the spectrum on Fano resonance. Therefore, in this paper, an analysis is carried out to investigate the propagation features of the proposed structure with respect to the geometrical parameters, which include the outer radius of the ring, the rotation angle of the internal h-shape, the length of the vertical rectangle of the internal h-shape of the ring, and the coupling distance between the annular cavity of the internal h-shape and the MIM waveguide.

## Materials and methods

**Fig 1:**
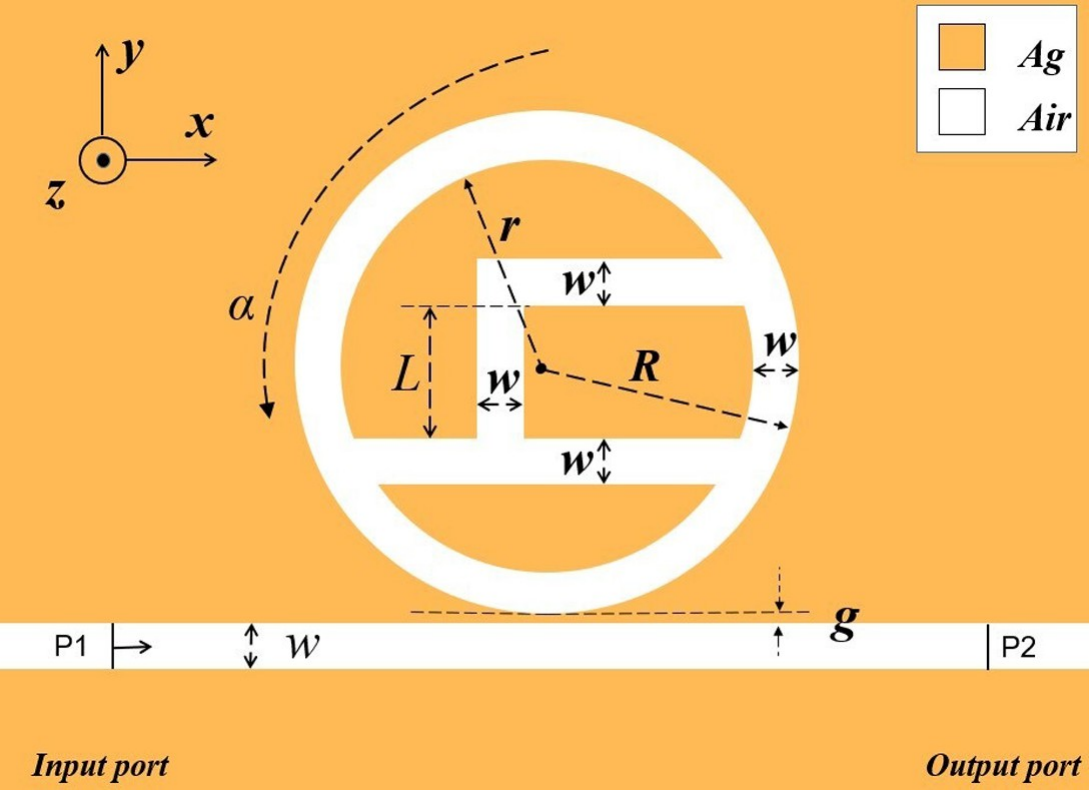
2D view of the designed sensor structure.

**Fig 2:**
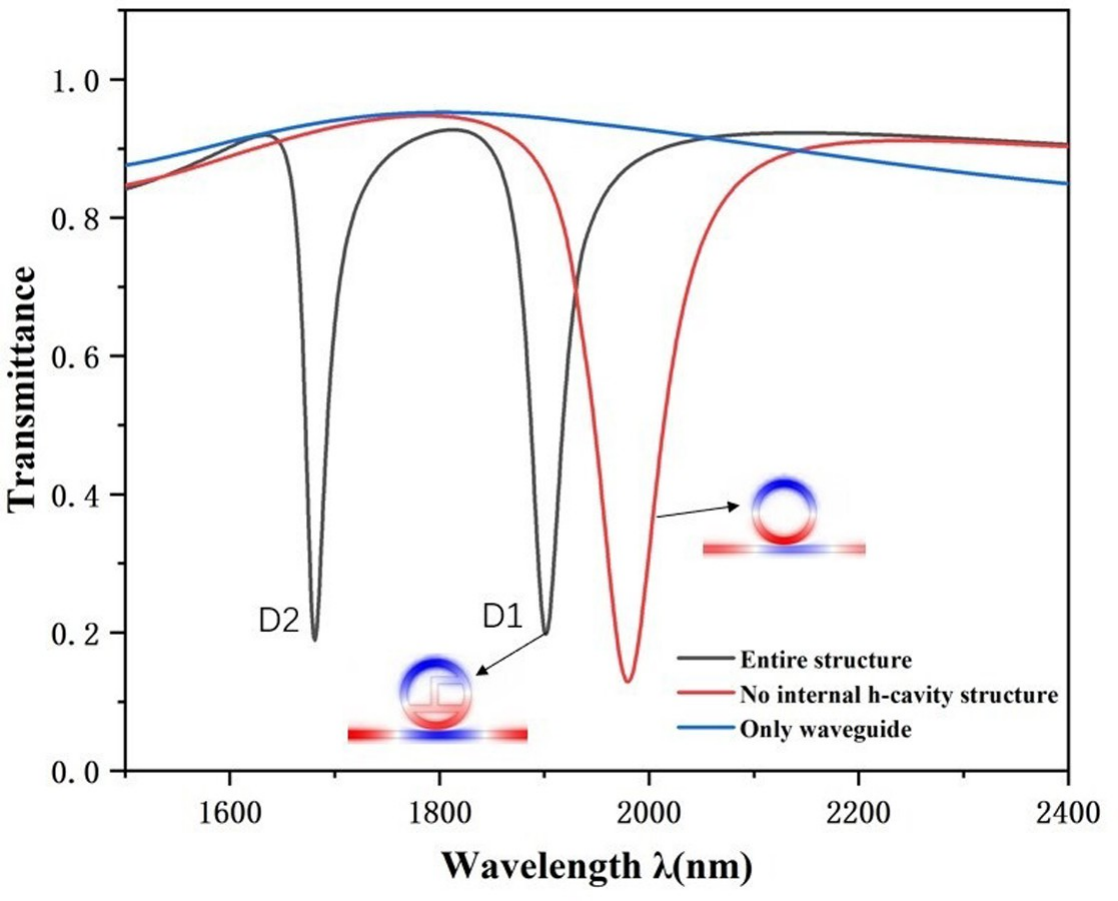
Transmission spectrum of the entire construction, structure without h-shaped cavity and waveguide-only structure.

Due to the fact that the thickness of the proposed architecture is far greater than the skinning width of the SPPs, the 3D phantom can be replaced by the 2D phantom for simple evaluation [26], and the error of such calculation is very small, which also reduces the computational pressure on the server and saves the computational time. The two-dimensional structure of the designed waveguide-coupled resonant cavity system is schematically shown in Fig 1, which consists of a ring resonant cavity having an h-shaped chamber and a MIM wire guide. The internal h-shaped cavity structure designed in this paper, firstly, the h-shaped structure can make the light wave energy more concentrated in the core region of the waveguide, reducing the unnecessary interaction with the surrounding medium, which helps to improve the transmission efficiency and stability of the waveguide. Secondly, the h-shaped structure effectively reduces the loss of light waves during transmission and enhances the coupling efficiency of the waveguide for specific frequency light. Finally, the h-shaped structure is relatively simple, which facilitates integration and fabrication at the micro- and nanoscale. Its geometry is clear and can be precisely fabricated using existing micromachining techniques. In addition, it was illustrated in the study that the sensors with internal h-shaped structural cavities also have higher sensitivity and better FOM. Compared to the simple circular cavity, as shown in Fig 2, comparing the normalised magnetic field distributions of the two structures, it is clear that the magnetic field of the inner h-shaped structure is deepened in colour, which means that its field strength is increased. Compared to complex structures, h-shaped structures are relatively simple and do not require much design optimisation to achieve good performance. This helps to reduce manufacturing costs and increase production efficiency. In addition, simple structures are also more conducive to theoretical analysis and simulation. In Fig 1, R and r are the external and internal radii of the circumference of the ring and satisfy R = r+50nm, L denotes that length of the vertical matrix of the h-shaped cavity inside the ring, α denotes the angle of counterclockwise rotation of the h-shaped cavity around the center, g is the distance between the RCIhS structure and the MIM waveguide, w denotes the widths of the ring cavity, the h-shaped cavity, and the MIM waveguide, and P1 and P2 denote the incoming and outgoing ports, respectively. The transmission distance of SPPs is shorter and accompanied by higher transmission loss in the odd-symmetric case, and longer and lower under the even-symmetric case [27]. When the coupling spacing g is larger, the two metal-dielectric interfaces are farther apart, so the coupling effect of SPPs will not be generated; when the value of the coupling spacing g is smaller, the SPPs generated at the two metal-dielectric junctions will be coupled mutually to form the parity-symmetric mode. In order for SPPs to have only even-symmetric modes in MIM waveguide propagation, the thickness of the dielectric layer w needs to be set to 50 nm [28–30]. Throughout this paper, w is set to 50 nm.

SPPs can be excited due to the specificity of the negative dielectric constant of metallic materials. Silver has a wide range of applications in optics, such as mirrors and transparent conductive films. In these applications, the stability of silver is critical to optical performance. Generally speaking, silver has better optical stability, and it is also not easy to corrode and has low energy loss. In order to better excite SPPs in MIM waveguide-coupled resonant cavity systems, we choose silver as the metallic material. It is shown in Fig 1 with the yellow portion representing silver and the white portion representing air. The Debye-Drude dispersion equation was used to describe the dielectric constant dispersion model of metallic silver, and the simulation was carried out using this equation, and the Debye-Drude dispersion equation is shown in Eq (1) [31]:

$$\varepsilon (w)=\varepsilon_{\infty }+\frac{\varepsilon_{s}-\varepsilon_{\infty }}{1+jw\tau }+\frac{\sigma }{jw\varepsilon_{0}}$$ (1)

where ε_*s*_ = -9530.5 is the quasi-static medium constant, ε_*∞*_ = 3.8344 is the relative permittivity at infinite angular frequency, ε_0_ is the permittivity in vacuum, $\tau =7.35\times 10^{-15}s$ is the relaxation time, and $\sigma =1.1486\times 10^{7}S/m$ is the conductivity of silver.

The equation for the transverse magnetic mode of the MIM waveguide is shown below [32, 33]:

$$tanh(kw)=\frac{-2kp\alpha_{c}}{k^{2}+p^{2}\alpha_{c}^{2}}$$ (2)

where *w* represents the waveguide width, *k* is the wavevector in the waveguide, $\alpha_{c}=\left[k_{0}^{2}\left(\varepsilon_{in}-\varepsilon_{m}\right)+\right.k{]}^{1/2}$, *p* = ε_*in*_/ε_*m*_. The wave vector *k*_0_ in vacuum is expressed as *k*_0_ = 2π/*λ*_0_. *k* is determined by Eq (2), and ε_*m*_ and ε_*in*_ denote the permittivity of the metal and the dielectric, respectively.

The simulation and analysis of the MIM waveguide-coupled resonant cavity system in this document are completed with the help of COMSOL, and the specific modeling and simulation steps are presented as follows. (1) Determine the appropriate physical field module and interface. In this paper, we design in two-dimensional space, choose the physical field module as the RF module, after which the corresponding electromagnetic field frequency domain is added to the physical field to complete a good basic environment; (2) Build the geometric model. The design structure can be drawn using COMSOL’s own geometric drawing tools; (3) Choose to set up the port, material model, and fluctuation equations and perform meshing. After the design structure is drawn, the corresponding materials are selected in the corresponding regions. In this paper, air is chosen as the filler material in the waveguide and resonant cavity, and silver is chosen as the filler material for the rest of the remaining parts. Set the left port as the incoming port and the right port as the outgoing port, and set the excitation of the outgoing port as off. The upper and lower boundaries of the MIM waveguide are set as perfectly matched layers as absorption boundary conditions. Finally, triangular meshing is performed on the design structure; (4) Simulation calculation. In the solver that comes with COMSOL, set the simulation range to 1500nm-3000nm and the simulation step to 1nm, and then click Calculate to perform the parameterized scanning; (5) The transmission spectra and field distributions of the MIM waveguide-coupled resonant cavity system can be obtained by performing the calculations in the above steps; (6) Results processing.

We evaluate the capability of this refractive index transducer using both sensitivity (S) and figure of merit (FOM) parameters in this paper. As a key indicator for appraising the performance of refractive index sensors, sensitivity (S) is defined as the linear trend of the resonance valley position in a MIM waveguide-coupled resonant cavity system as the ambient refractive index is changed. The sensitivity can be characterized by Eq (3) [34]:

$$S=\frac{\Delta \lambda }{\Delta n}$$ (3)

where Δ*n* is the variation of the ambient refractive index and Δ*λ* is the modification of the resonance length that occurs as ambient refractive index changes. The unit of sensitivity S is expressed as nm/RIU.

FOM is also an influential metric used to characterize the transducer, and the proposed structure needs to be parameterized in the experiments to obtain an optimal FOM value, which is expressed by the formula [35]:

$$FOM=\frac{S}{FWHM}$$ (4)

where FWHM denotes the half height width, which is the width of the spectral line at half the height of the curve. When comparing FOM results from different research works, different studies may use different definitions of FOM. This difference mainly stems from the different physical quantities and application contexts that different studies focus on. Different definitions are in essence used to assess the merit of a certain performance or effect. In this work, we adopt the ratio of sensitivity (S) to half width height (FWHM) as the definition of FOM.

## Simulation results and analysis

First we plotted the transmittal curves for the entire construction, the construction having no h-shaped cavity, and the waveguide-only structure. As shown in Fig 2, the black curve represents the Fano resonance curve for the complete structure, the red color represents the transmission curve without the internal h-shaped cavity structure, and the blue color represents the transmission curve with the MIM waveguide only. From the blue MIM waveguide curve we can see the transmissive shape is nearly flat with a massive continuum zone, which we refer to the broad bandwidth pattern of the Fano resonance. Nevertheless the rest of the two curves both have variable inclined locations.

**Fig 3:**
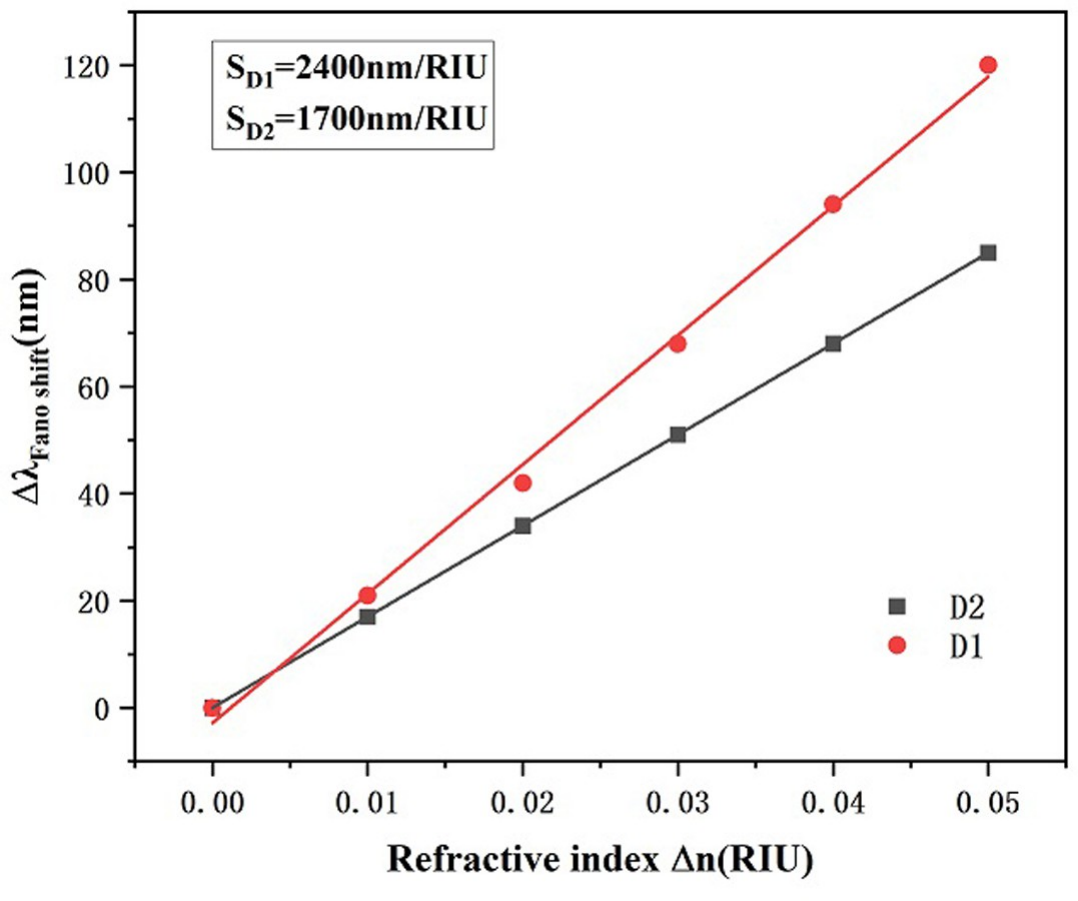
Sensitivity fit at D1 and D2.

We further compare and analyze Fig 2, in which we compare the black and red transmit waveforms, and evidently we observe that the waveforms have altered remarkably. The number of wave valleys of the overall structure increases significantly, and there is a Fano resonance splitting, which increases from one to two wave valleys, that is, D1 and D2, as shown in Fig 3 it can be seen that the sensitivity at D1 is higher than that at D2, and there is not a big difference in the FWHM values, and thus in this paper, we choose D1 for the next step of the study. The overall shift of the transmission curve to the left with the addition of the h-shaped cavity indicates a slight decrease in the sensitivity of the system. However, the FWHM values of the transmission curves are smaller with the addition of the h-shaped cavity structure. Comparing the normalized magnetic field distributions of the two structures, it is obvious to see that the color of the electric velocity of the RCIhS system is deepened, which means that its field strength increases. Thus although the sensitivity of the RCIhS structure is slightly reduced, the quality factor FOM of the RCIhS structure is ultimately improved because of the significant reduction in the FWHM value.

**Fig 4:**
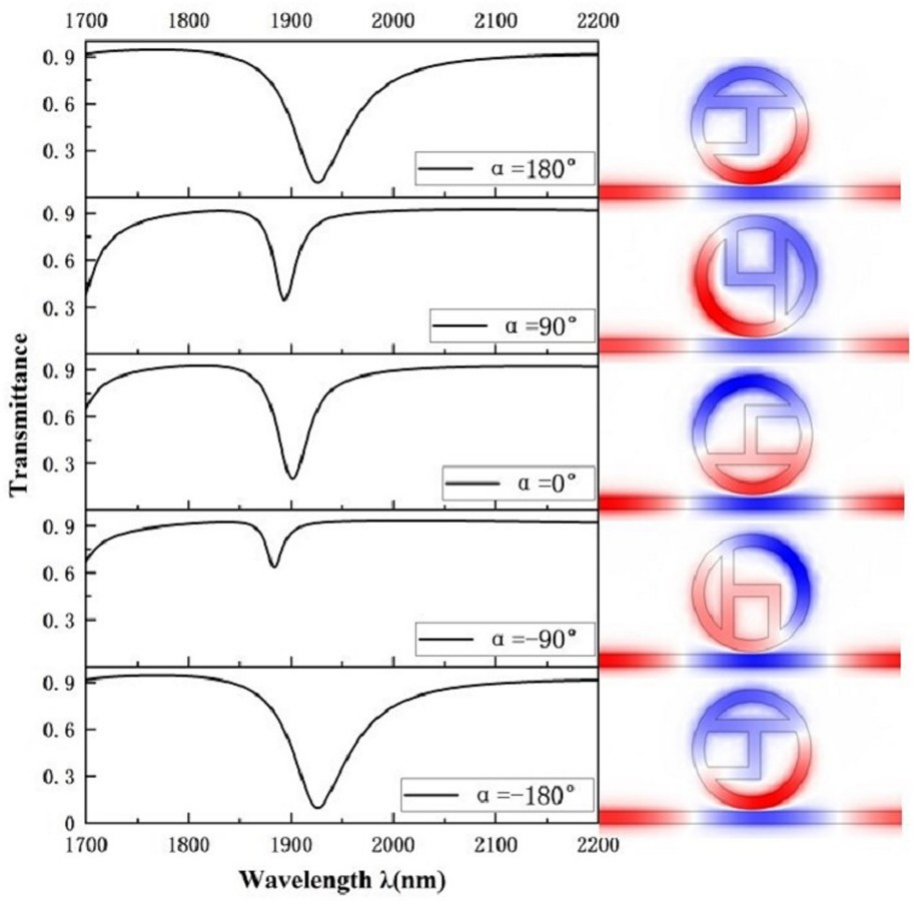
Transmission spectrum of h-shaped cavity rotating at different angles.

We study the effect of structural geometric parameters on the transmission characteristics of waveguide-coupled resonant cavity systems. Based on the above discussion of the formation of the Fano resonance phenomenon, we learned that the addition of the h-shaped chamber is the essential structure for formation of the Fano resonance curve. Next, in order to evaluate the effect of the rotation angle of the h-shaped cavity on the propagation characteristics, the geometrical parameters were set to R = 250 nm, r = R-50 nm, L = 150 nm, α = 0°, and g = 10 nm, and the rotation angle of the h-shaped cavity was varied by setting the rotation angles to -180°, -90°, 0°, 90°, and 180°. The transmission spectra corresponding to the five derived structures are presented in Fig 4, where it can be seen that the h-shaped cavity divides the circular cavity into two parts with opposite phases of the magnetic field. The h-shaped cavity has the same structural diagram when rotated 180° around the counterclockwise and clockwise directions, which produces a similar transmission spectral profile, which is shifted to the right compared to the one at 0° and has a lower transmittance, indicating that this structure has a better ability to confine the incident light. However, it can be seen from Fig 4 that the FWHM value of the curve at 180° is significantly larger than 0°, and from Eq (4), the larger the FWHM, the smaller the FOM. When the h-shaped cavity was rotated by 90° in counterclockwise and clockwise directions, different structural maps were produced, and different transmission spectral curves were formed, but compared with the transmission spectral curves at 0°, their transmittances both became significantly higher, and the higher transmittances indicated that the two derived structures were less capable of limiting the incident light. The transmission spectrum at α of 0° is an unsymmetrical shape having an extra-low permeability and a great wavelength, suggesting that this is the time of the Fano resonance with a remarkably sensitive nature. Thus, we choose the structure when α is 0° for the next study.

**Fig 5:**
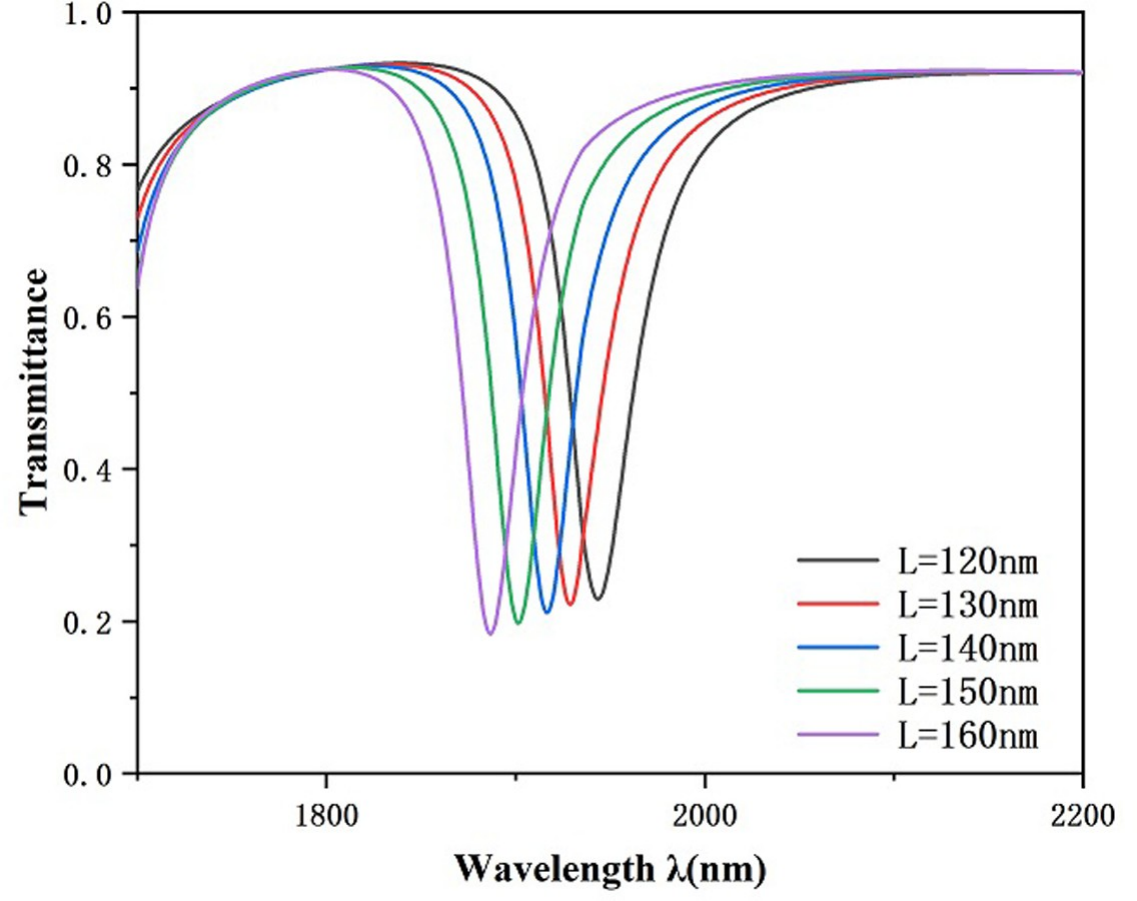
Transmission spectrum of h-shaped cavity with vertical rectangle of different lengths.

**Fig 6:**
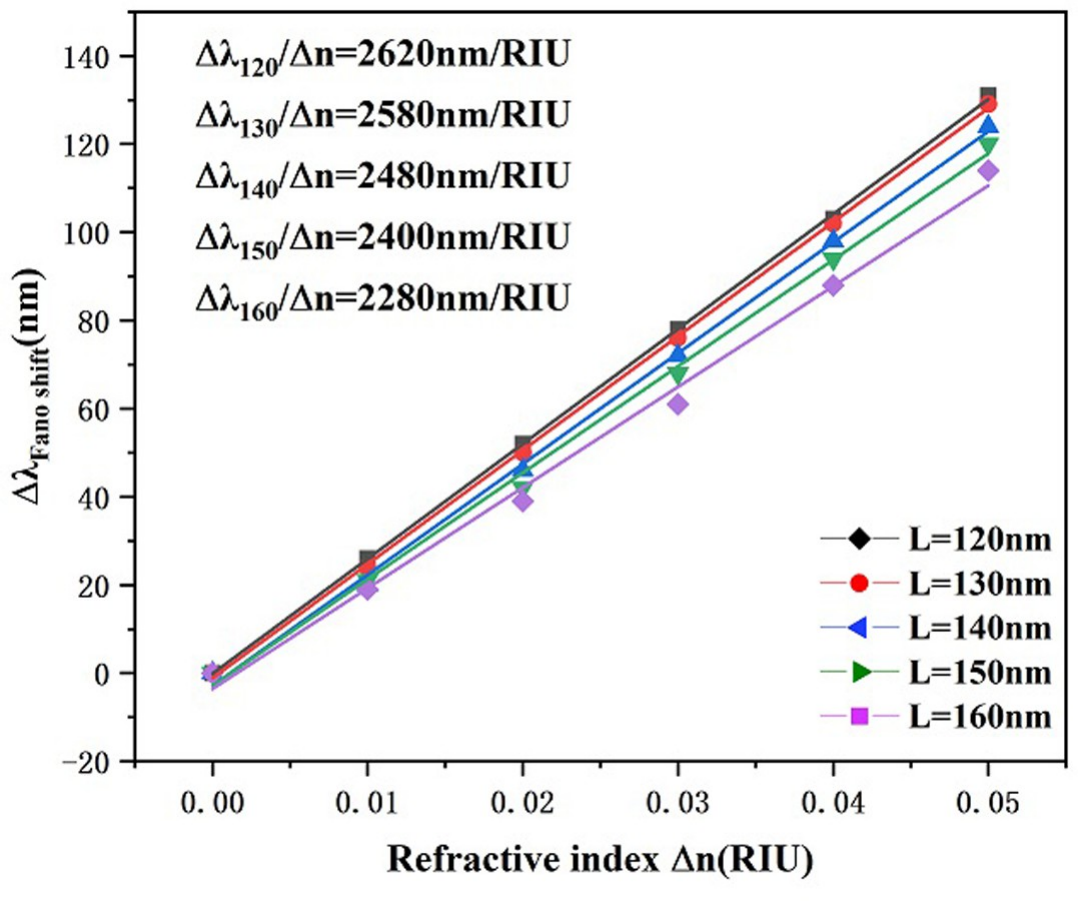
Sensitivity fitted lines of vertical rectangles of different lengths L for h-shaped cavities.

**Fig 7:**
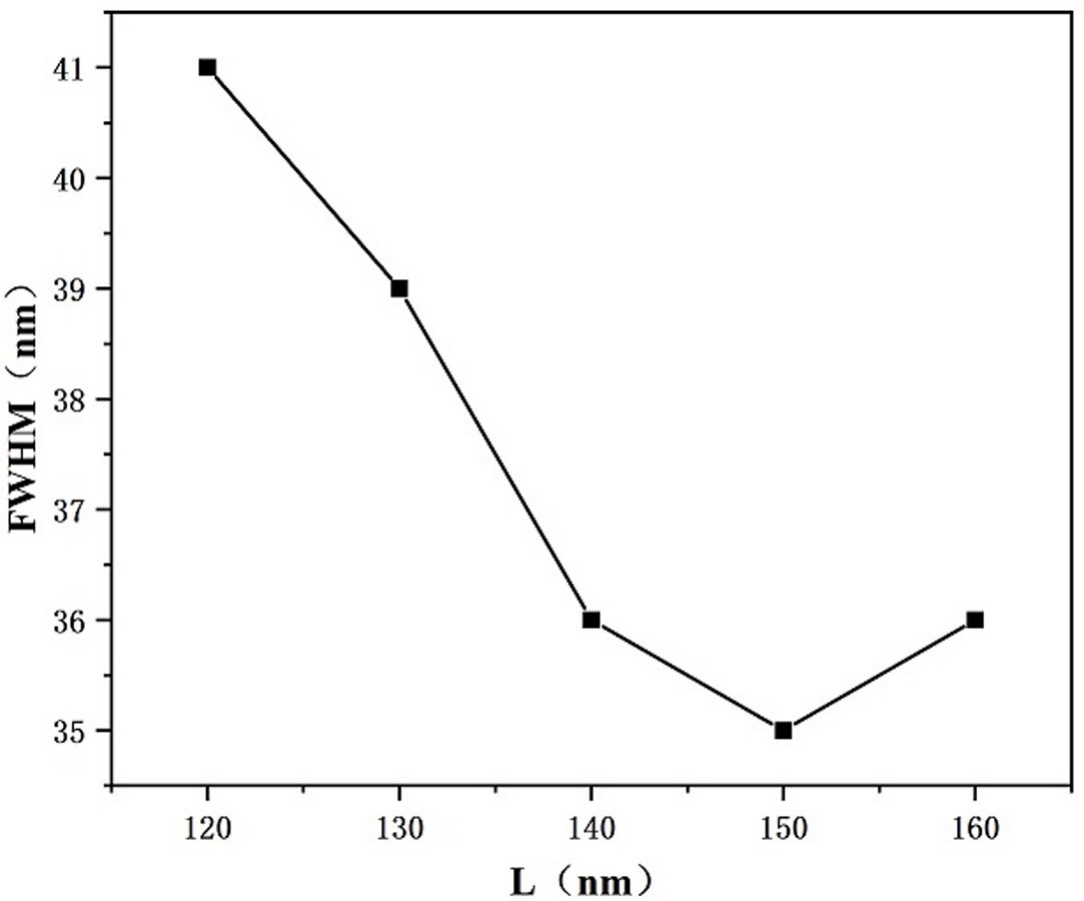
Variation of FWHM values for different lengths L of the vertical rectangle of the h-shaped cavity.

In a further step we investigate the effect of inwardly connected h-shaped cavity’s vertical rectangle’s longitudinal length L on the transmission characteristics. Other parameters were kept constant, and the lengths of the vertical rectangle of the internally connected h-shaped cavity was set to 120 nm, 130 nm, 140 nm, 150 nm, and 160 nm to study its effect on the Fano resonance transmitted spectrum. There is an overall movement of the curve to the left with the increase of L and a slight decrease of the transmittance as indicated in Fig 5, which shows that larger L has a better ability to capture the SPPs. As shown in Fig 6, the sensitivity decreases from 2620 nm/RIU to 2280 nm/RIU as L increases. From Fig 7, it can be seen that the corresponding FWHM value is the smallest when L is 150 nm, while the corresponding FWHM value is the largest when L is 120 nm, thus there is a better FOM when L is 150 nm and the transmittance is lower when L is 150 nm, which allows for better capture of SPPs. Considering all the factors, in this paper we choose L to be 150 nm for the subsequent study, at which time the whole system has suitable sensitivity and FOM.

**Fig 8:**
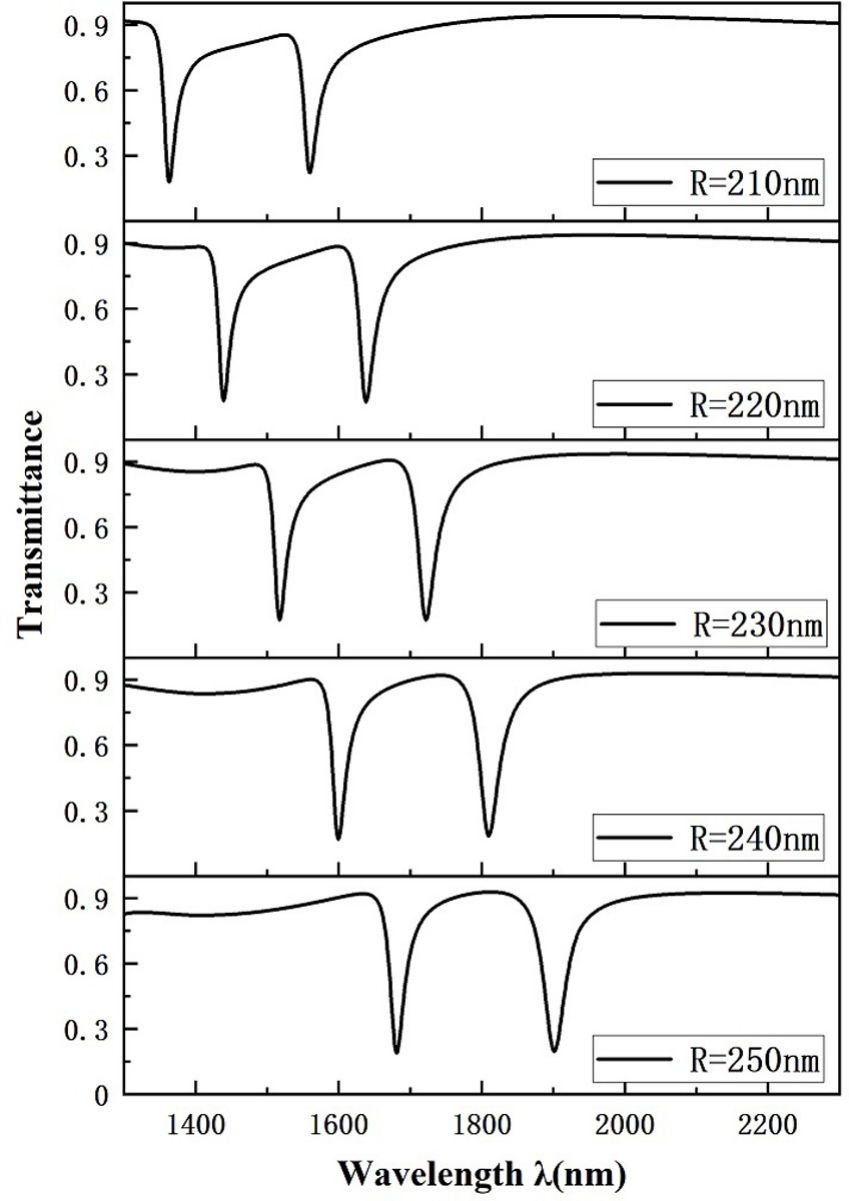
Transmission spectrum of a circular circle with variable outer radius R.

**Fig 9:**
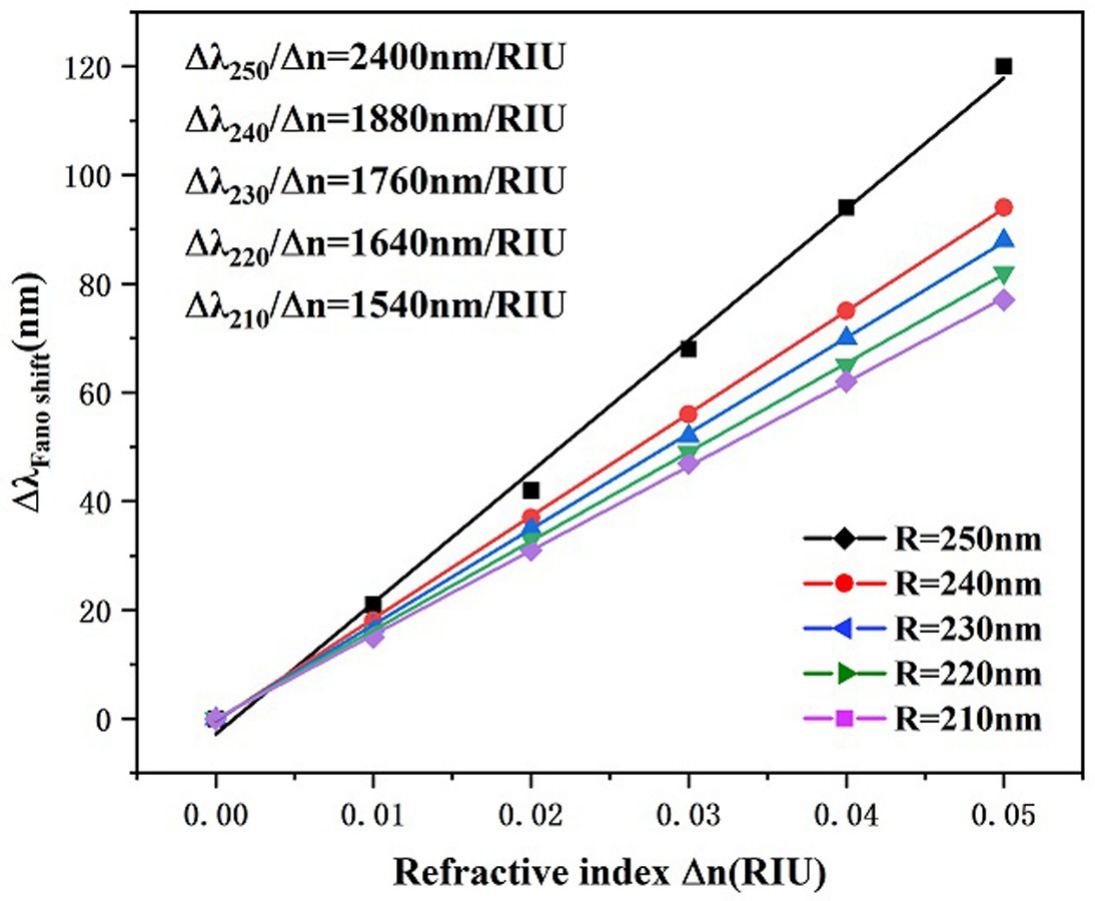
Sensitivity fit lines for rings with different outer radii R.

Subsequently, we investigated the variation of the outer radius value R of the circle. Ensuring that the other geometric parameters of the system remain unchanged, the value of the external radiator R of the circle is assumed to be set to 210 nm, 220 nm, 230 nm, 240 nm, and 250 nm. The transmission spectra due to the variation of the outer radius of the resonant cavity are plotted in Fig 8. With increasing outer radius, the entire Fano resonance curve undergoes a significant rightward shift, which arises from the growth of the effective thickness of the resonant cavity, resulting in a shift of the resonance trough towards larger wavelengths. In addition, we can see that the curved transmittance decreases as the outer radius of the circle increases. Fig 9 represents the sensitivity fitting curves for different outer radii of the system, showing a strong linear fit. From the figure, it is observed that the sensitivity of the proposed model is greatly improved from 1540 nm/RIU to 2400 nm/RIU with the expansion of the outer radius R. Thus, an outer radius of the circle R is the key factor used to increase the sensitivity of the structure, and in practice, the adequate dimension can be elected based on the equipment sensitivity manufacturing demands used. In this paper we choose an outer radius of 250 nm for our study.

**Fig 10:**
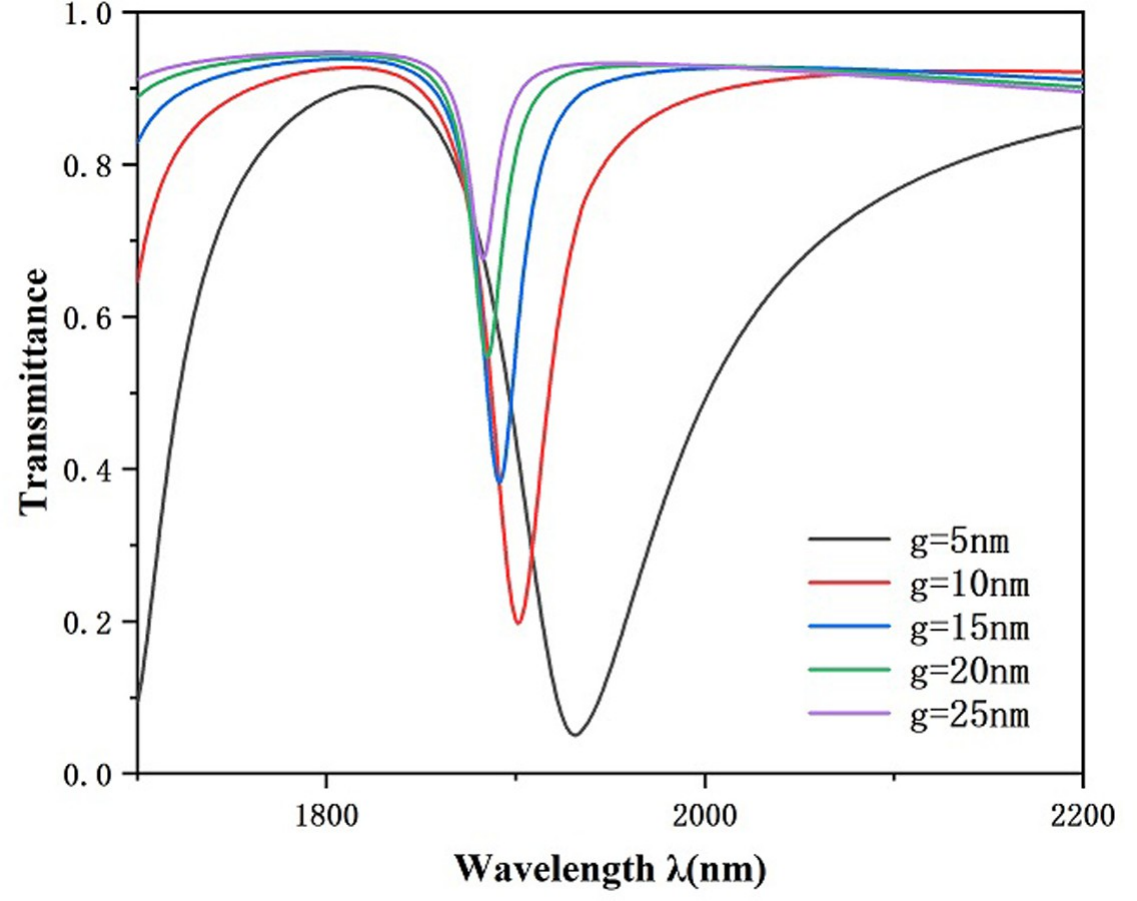
Transmission spectrum for variable coupling distances g.

**Fig 11:**
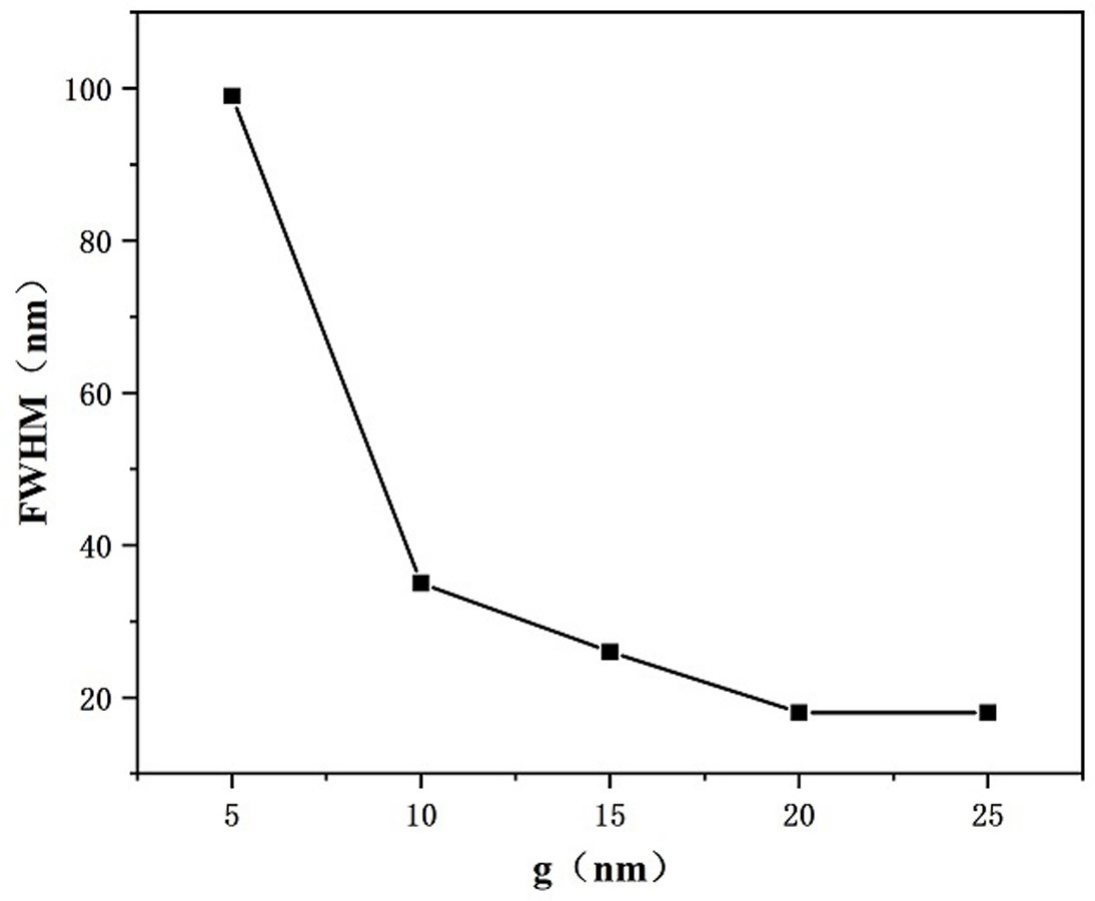
Variation of FWHM with variable coupling lengths g.

**Fig 12:**
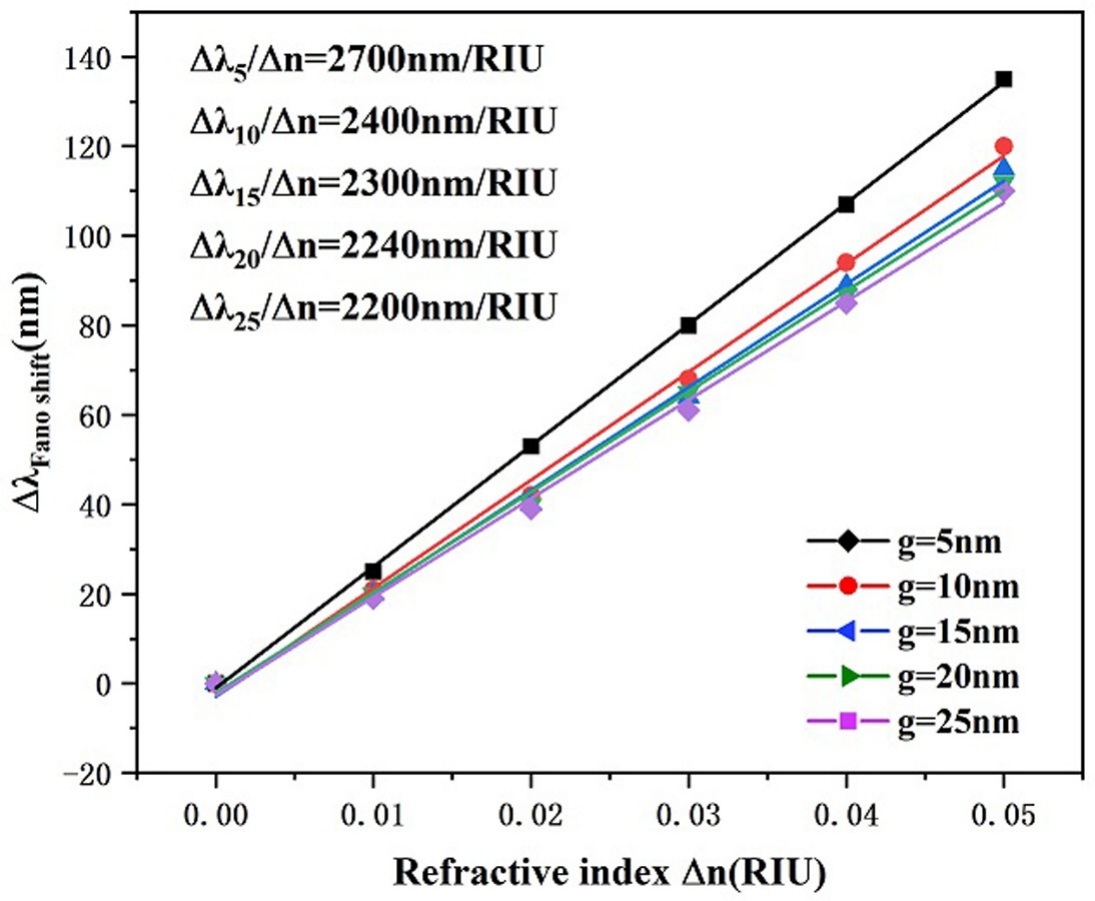
Sensitivity fit lines for different coupling distances g.

Finally, we investigated the effect of the bonding distance g between MIM waveguide together with the RCIhS construction on the sensing features of the system by setting the coupling distance g to 5 nm, 10 nm, 15 nm, 20 nm, as well as 25 nm. As shown in Fig 10, the transmittance spectral curve of the system undergoes a significant left shift and the transmittance increases when the coupling distance g is increased, in addition the FWHM value decreases with the coupling distance. This shows that the coupling between the SPPs in the MIM waveguide and the resonant cavity of the RCIhS structure becomes more difficult as the coupling distance increases, and the electric field strength becomes weaker with the increase of the coupling gap, resulting in a decrease in the sensing performance of the system, which suggests that sensing profiles with a low transmittance can be obtained by choosing a suitable coupling distance g. From Fig 11, it can be seen that when the coupling distance g is 5 nm, the corresponding FWHM value is the largest, which is 99 nm, and the FOM value at this time is 27.27 from Eq (4); when the coupling distance is larger than 10 nm, the FWHM value decreases with the coupling distance, but at the same time, the transmittance also increases sharply. From Fig 12, it can be seen that the sensitivity does not vary much with the coupling distance when the coupling distance is greater than 10 nm. When the coupling distance is smaller than 10 nm, significantly higher FWHM spoils the FOM of the architecture. After comprehensive consideration, in this paper we choose 10 nm for the preferable coughing distance of the presented transducer.

**Fig 13:**
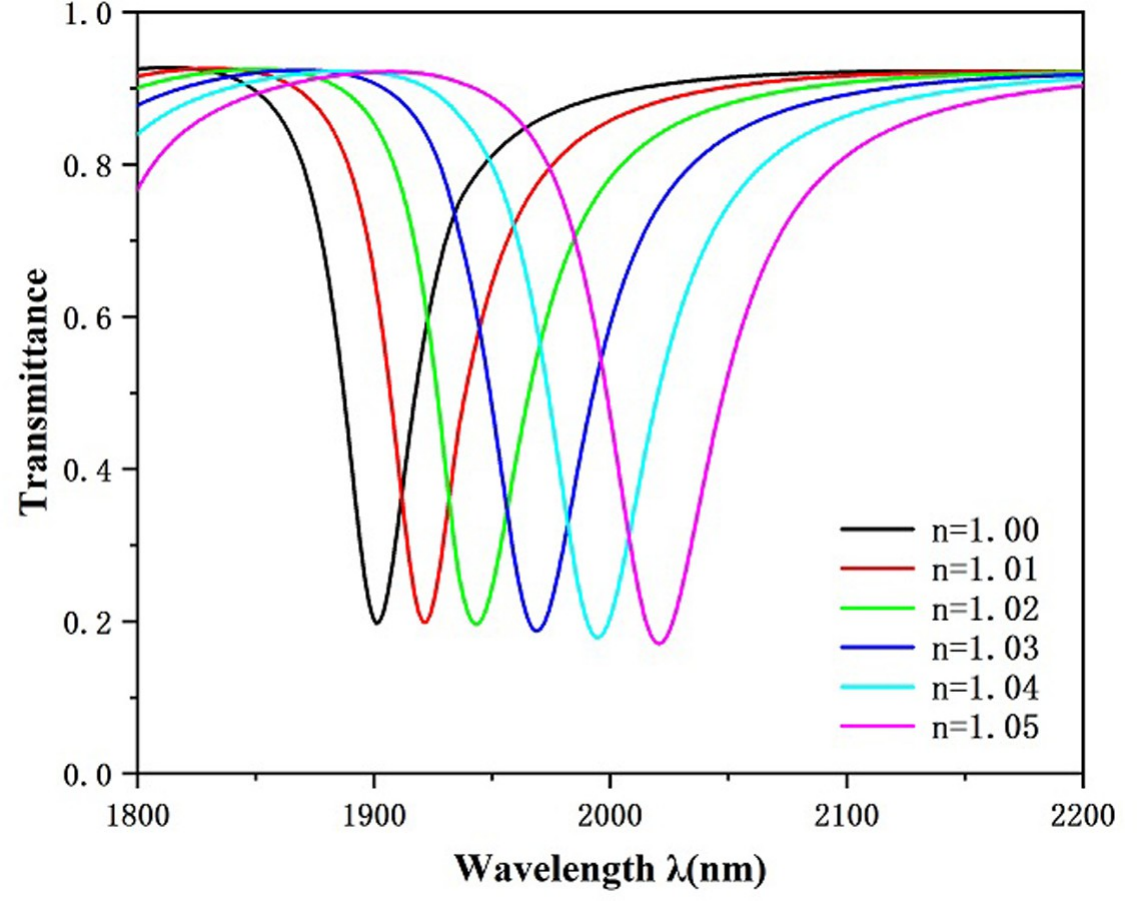
Transmission spectrum for different values of refractive index n.

**Fig 14:**
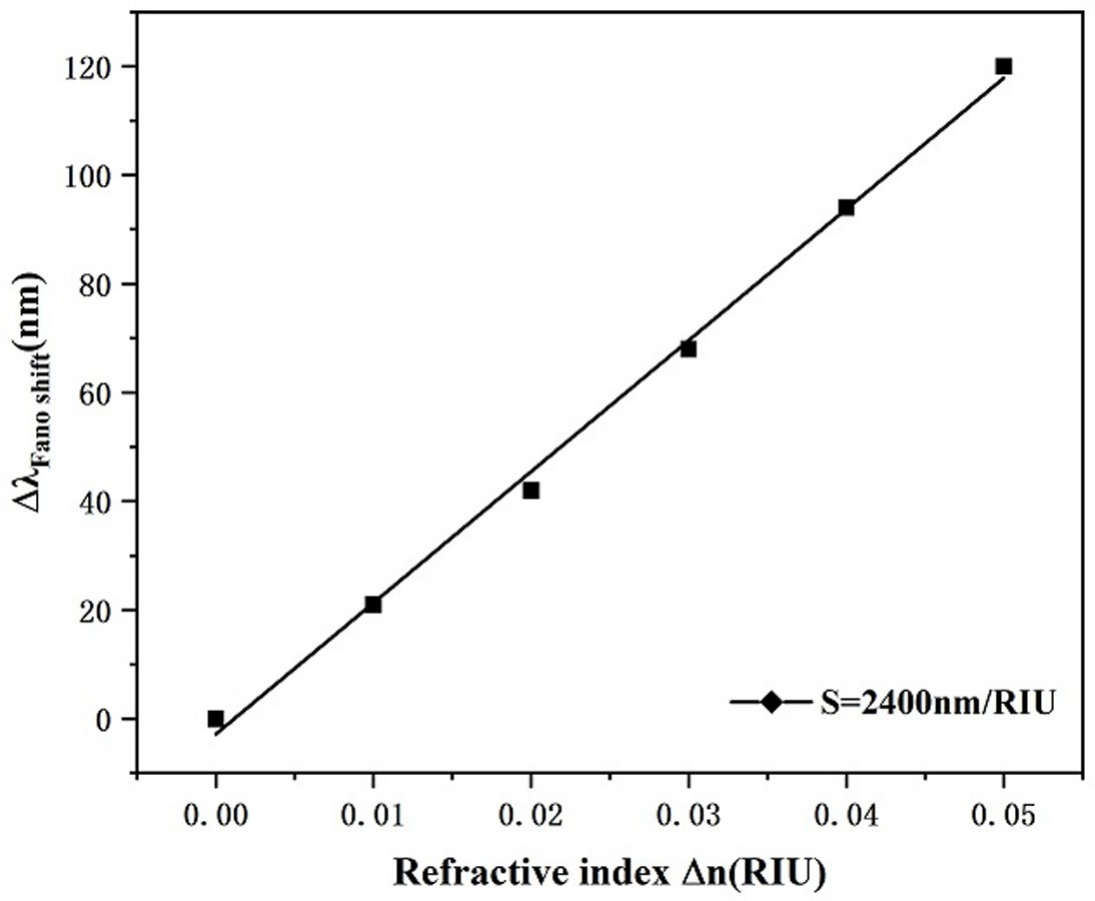
Sensitivity fit lines for different refractive indices n.

According to the studies on the geometric features of the system structure presented above, in order to realize the best sensing performance of the waveguide-coupled resonant cavity system, we set the geometric features of the system structure as follows: α = 0°, L = 150 nm, R = 250 nm, and g = 10 nm. Next, we investigate the difference of reflectivity change on the sensor performances of the system by setting the refractive index to 1.00, 1.01, 1.02, 1.03, 1.04, and 1.05 for the study and go to evaluate the sensing performances of the system as the refractive index is changes. As shown in Fig 13, the transmission spectra are plotted for different refractive indices n. From the figure, we can see that there is an equidistant rightward shift in the transmission spectral curve as the refractive index increases. Thus, we can calculate the sensibility of the designed transducer depending on the shifting range of the resonance trough. As shown in Fig 14 is the sensitivity fit of the system, from which it can be seen that the highest achievable is 2400 nm/RIU, which corresponds to a FOM of 68.57. This is better than the available data given in Table 1.

**Table 1:** Comparisons of the results with extant studies.

References	Structure Type	Sensitivity(nm/RIU)	Figure of Merit
[36]	A metal-insulator-metal based arc-shaped resonator coupled with a rectangular stub	1900	23.75
[37]	The metal-insulator-metal waveguide with a stub coupled with a split-square resonator	1125.7	30.01
[38]	Asymmetric rectangular periodic nanostructured sensors	419.45	-
[39]	MIM waveguide-coupled double rectangular cavities	596	-
This work	RCIhS structure	2400	68.57

## Application

Due to the high sensitivity, simple structure and high quality factor of the above proposed sensors, they can be used as temperature sensor applications. Because the refractive index-temperature correlation coefficient of ethanol (3.94×10^−4^) is two orders of magnitude higher than the refractive index-temperature correlation coefficients of the silver (9.30×10^−6^) and quartz (8.60×10^−6^) materials, we chose ethanol as the sensitive medium material. Thus, when ethanol is filled into the RCIhS structure, the refractive index inaccuracy caused by the peripheral temperature goes unnoticed. The ethanol has a 78°C and -144°C boiling and mid-range melting points, approximately, in which the linearity between the ethanol’s thermal and refractive indices can be expressed as [40]:

$$n=1.36048-3.94\times 10^{-4}\left(T-T_{0}\right)$$ (5)

where T represents the environmental temperature while T_0_ denotes the indoor temperature, and its value is established at 20°C.

Furthermore, considering the fusion point and zeal point of ethanol, we set our test temperature ranging from -90°C to 70°C. The structural values of the transducer were parameterized as α = 0°, L = 150 nm, R = 250 nm, and g = 10 nm. The sensitivity of the temperature sensor is expressed as:

$$S_{T}=\Delta \lambda /\Delta T$$ (6)

where Δλ denotes the displacement of the transmitted light spectrum as well as ΔT denotes the amount of temperature variations.

**Fig 15:**
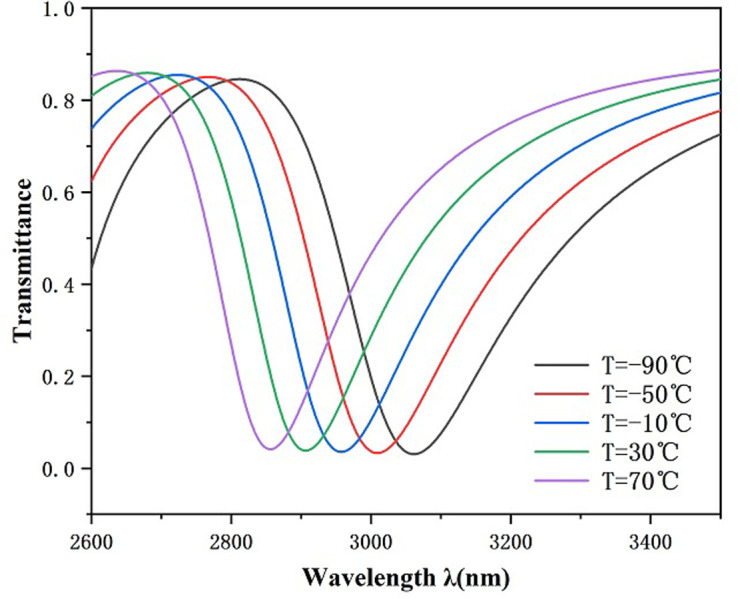
Transmission spectrum for temperature change.

**Fig 16:**
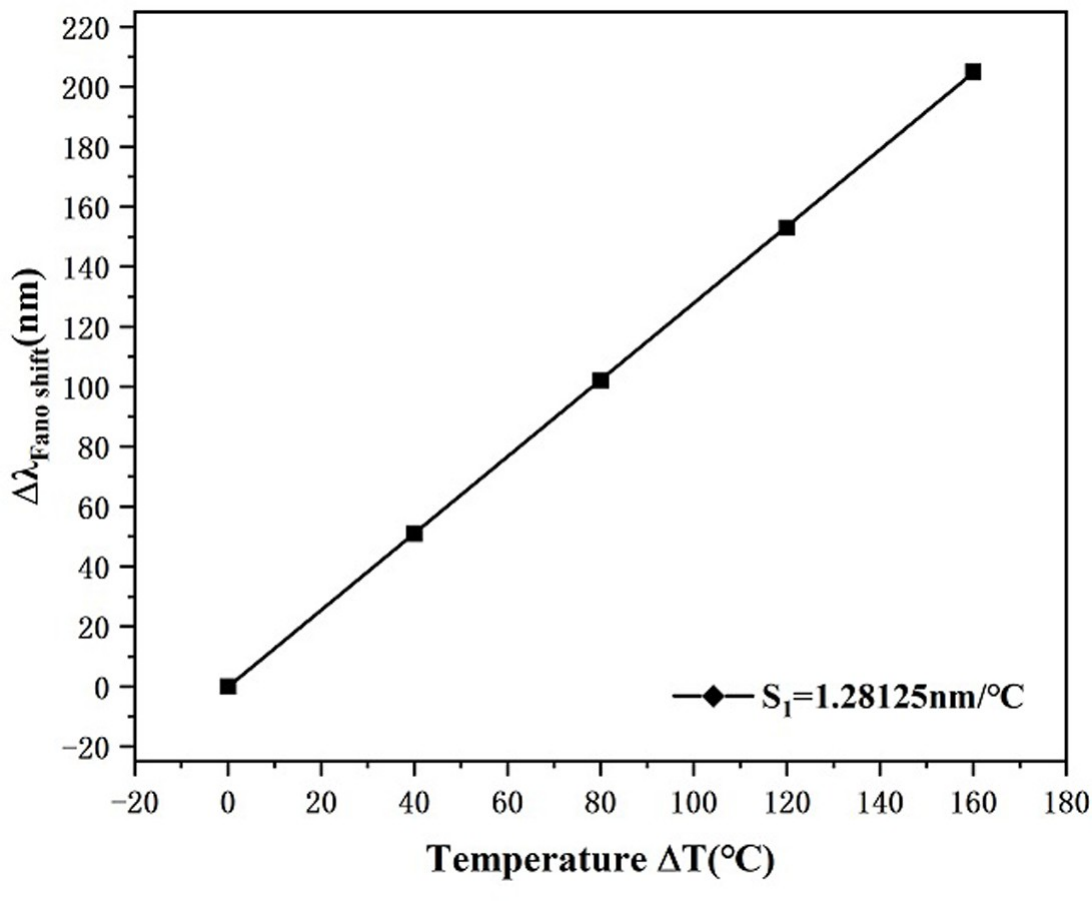
Temperature sensor sensitivity fitting plot.

From Eq (5), it can be obtained that the refractive index of this sensor changed from 1.40382 to 1.34078 in the set temperature range. Fig 15 shows a noticeable left movement of the transmission spectrogram with rising temperature, and the position of the resonance trough changes from 3061 nm to 2856 nm, that is, Δλ is 205 nm. Fig 16 represents the sensitivity fit line for this temperature sensor, and the good linear fit of the curve ensures the precision of the estimation. After calculation, the S of this temperature transducer was obtained as 1.28125 nm/°C. This is better than some of the available data listed in Table 2.

**Table 2:** Comparison of temperature sensor performance.

References	Temperature sensitivity(nm/°C)
[41]	1.00
[42]	0.22
[43]	0.5
This work	1.28125

## Conclusions

Here we have presented a novel transducer construction for waveguide-coupled resonant cavities, which is composed of a MIM waveguide and an annular cavity with an internal h-shaped cavity. A series of experimental comparisons were carried out using COMSOL software and the FEM to investigate and discuss the spread characteristics of the system construction, which led to the determination of the optimal structural parameters and the acquisition of an excellent Fano resonance curve. The results show that the sensitivity of the designed sensor depends mainly on the outer radius R of the resonant cavity. The wavelength of the Fano resonant dip shifts significantly to the right by increasing the refractive index, n, and the outer annular radius, R, while increasing the coupling distance results in a significant left shift of the Fano resonant dip wavelength. A larger effect of the rotating angles of the internally connected h-shaped cavities on the Fano resonance inclination wavenumber and transmittance is observed. The optimum sampling characteristics of the suggested sensor are optimized when the geometrical parameters of the structure are α = 0°, L = 150 nm, R = 250 nm, and g = 10 nm, and the transducer exhibits a sensitivity of 2400 nm/RIU and a FOM of 68.57. In conclusion, this paper describes the usage of this architecture for the application in the sphere of temperature sampling, which can go up to a sensitivity of 1.28125 nm/°C. This provides a certain reference value for the future research of integrated optical sensor devices, and greatly promotes the exploration of the field of miniaturized optical sensors.
